# Supplementary material for: Initial mean arterial blood pressure (MABP) measurement is a risk factor for mortality in hypertensive COVID-19 positive hospitalized patients
Source: PLoS One. 2023 Mar 30;18(3):e0283331. doi: 10.1371/journal.pone.0283331 (PMC10062544; doi:10.1371/journal.pone.0283331)
Supplement: S1 Table — (DOCX) [file pone.0283331.s001.docx]

**Supporting Information**

**S1 Table. Variables measure in COVID-19(+) and COVID-19(-) hospitalized patients**

|  | **Variables** |
| --- | --- |
|  |  |
|  | **Demographics** |
| 1 | Age (years) |
|  | **Gender** |
| 2 | Female |
|  | Male |
| 3 | **Race** |
|  | American Indian or Alaska Native |
|  | Asian |
|  | Black or African American |
|  | Other Race/Unknown |
|  | White |
| 4 | **Ethnicity** |
|  | Hispanic or Latino |
|  | Not Hispanic |
|  | **Comorbid conditions** |
| 5 | diabetes_mellitus |
| 6 | HF (heart failure) |
| 7 | CKD (chronic kidney disease) |
| 8 | COPD (chronic obstructive pulmonary disease) |
| 9 | HTN (hypertension) |
| 10 | CAD (coronary artery disease) |
| 11 | Cancer |
| 12 | Asthma |
| 13 | Suicidal Thoughts |
| 14 | Major Depression |
| 15 | Schizophrenia |
| 16 | Bipolar |
| 17 | ADHD (attention deficit hyperactivity disorder) |
| 18 | Anxiety |
| 19 | BMI (Body Mass Index) |
|  | **Severity of illness** |
| 20 | Length of Hospital Stay (days) |
| 21 | Invasive vent days (invasive ventilation) |
| 22 | ICU Admission |
| 23 | Length of ICU stay (days) |
| 24 | Sepsis |
| 25 | Vasopressor Indicator |
|  | **Medications** |
| 26 | Enoxaparin |
| 27 | Heparin |
| 28 | Wafarin |
| 29 | Rivaroxaban |
| 30 | Dabigatran |
| 31 | Argatroban |
| 32 | Hydroxychloroquine |
| 33 | Azithromycin |
| 34 | Dexamethasone |
| 35 | Salicylic_acid_and_derivatives |
| 36 | Ace inhibitor_plain |
| 37 | Arb |
|  | **Vitals** |
| 38 | SBP (systolic blood pressure, mm Hg) |
| 39 | DBP (diastolic blood pressure, mm Hg) |
| 40 | PPD (pulse pressure difference, mm Hg) |
| 41 | MABP (mean arterial blood pressure, mm Hg) |
| 42 | heart rate (beats/min) |
| 43 | oral temperature (^o^C) |
| 44 | respiratory rate (respirations/min) |
|  | **Respiratory Measures** |
| 45 | Pulse Ox (%) |
| 46 | PaO2 (partial pressure of arterial oxygen, mm Hg) |
| 47 | FiO2 (fraction of inspired oxygen, %) |
| 48 | Osmolality (serum osmolality, mosm/Kg) |
| 49 | pH_arterial |
| 50 | O2_arterial (mm Hg) |
| 51 | CO2_arterial (mm Hg) |
|  | **Renal Lab** |
| 52 | BUN (blood urea nitrogen, mg/dL) |
| 53 | Sodium (serum sodium, meq/L) |
| 54 | K (serum potassium, meq/L) |
| 55 | Cl (serum chloride, meq/L) |
| 56 | hco3 (serum bicarbonate, meq/L) |
| 57 | Ca (serum calcium, mg/dL) |
| 58 | Ca_ionized (ionized calcium, mg/dL) |
| 59 | Phosphate (mg/dL) |
| 60 | First Mg (magnesium) |
| 61 | Na urine (urine sodium, meq/L) |
| 62 | Osmolality urine (mosm/Kg) |
| 63 | Creatinine (serum creatinine, mg/dL) |
| 64 | Creatinine urine (urine creatinine, mg/dL) |
| 65 | Urea urine (urine urea, mg/dL) |
| 66 | Protein urine strip (urine protein by dipstick) |
| 67 | Protein Urine (spot urine protein, mg/dL) |
| 68 | RBC urine (urine red blood cells) |
| 69 | Renin (serum renin, ng/mL/hr) |
|  | **Inflammatory Labs** |
| 70 | Ferritin (mcg/L) |
| 71 | Albumin Serum (mg/dL) |
| 72 | Lymphocyte Count (K/mm^3^) |
| 73 | Procalcitonin (ng/mL) |
| 74 | D-Dimer (ng/mL) |
| 75 | IL6 (pg/mL) |
| 76 | WBC (10^9^ cell/L) |
| 77 | ESR (mm/hr) |
| 78 | CRP (mg/L) |
|  | **Other Labs** |
| 79 | HB (g/dL) |
| 80 | Lactate (mmol/L) |
| 81 | BNP (pg/mL) |
| 82 | Troponin (ng/mL) |
| 83 | INR |
| 84 | LDH (lactate dehydrogenase, U/L) |
| 85 | AST (aspartate aminotransferase, U/L) |
| 86 | ALT (alanine aminotransferase, U/L) |
| 87 | CPK (creatinine phosphokinase, U/L) |
|  | **Lipid Profile** |
| 88 | LDL (low density lipoprotein, mg/dL) |
| 89 | Triglyceride (mg/dL) |
| 90 | HDL (high density lipoprotein, mg/dL) |
| 91 | **death** |
|  |  |
