# Supplementary material for: Initial mean arterial blood pressure (MABP) measurement is a risk factor for mortality in hypertensive COVID-19 positive hospitalized patients
Source: PLoS One. 2023 Mar 30;18(3):e0283331. doi: 10.1371/journal.pone.0283331 (PMC10062544; doi:10.1371/journal.pone.0283331)
Supplement: S2 Table — (DOCX) [file pone.0283331.s002.docx]

**S2 Table. Tertiles of MABP in COVID-19 (+) patients**

| **Variables** | **COVID-19 Positive** | | | | |
| --- | --- | --- | --- | --- | --- |
|  | **T1 ≥65- <86 mmHg** | **T2# ≥86-<98 mmHg** | **T3 ≥ 98 mmHg** | **p- value^1^** | **p-value^2^** |
|  | N=504 | N=551 | N=452 |  |  |
| **Comorbid conditions** |  |  |  | 0.999  0.983  0.999  0.999 |  |
| Cancer | 46 (9.1%) | 40 (7.3%) | 26 (5.8%) |  | 0.999 |
| Asthma | 42 (8.3%) | 33 (6.0%) | 31 (6.9%) |  | 0.999 |
| Suicidal Thoughts | 6 (1.2%) | 5 (0.9%) | 2 (0.4%) |  | 0.999 |
| Major Depression | 76 (15.1%) | 76 (13.8%) | 62 (13.7%) |  | 0.999 |
| Schizophrenia | 10 (2.0%) | 11 (2.0%) | 8 (1.8%) | 0.999 | 0.999 |
| Bipolar | 15 (3.0%) | 15 (2.7%) | 8 (1.8%) | 0.999  0.999 | 0.999 |
| ADHD (attention deficit hyperactivity disorder) | 2 (0.4%) | 5 (0.9%) | 0 (0.0%) |  | 0.999 |
| Anxiety | 81 (16.1%) | 85 (15.4%) | 53 (11.7%) | 0.999 | 0.639 |
| BMI | 28.07 (6.85) | 28.13 (7.21) | 28.20 (7.28) | 0.999 | 0.999 |
| **Severity of illness** |  |  |  |  |  |
| Length of Hospital Stay | 8.00 (5.00, 13.00) | 8.00 (5.00, 13.00) | 7.00 (5.00, 14.00) | 0.999 | 0.999 |
| Invasive vent days (invasive ventilation) | 10.50 (5.00, 21.00) | 13.00 (8.00, 24.00) | 12.50 (7.50, 25.50) | 0.769 | 0.999 |
| ICU Admission | 124 (24.6%) | 101 (18.3%) | 95 (21.0%) | 0.093 | 0.999 |
| Length of ICU stay | 8.00 (3.00, 17.00) | 11.00 (6.00, 26.00) | 13.00 (5.00, 23.00) | 0.020* | 0.999 |
| Sepsis | 166 (32.9%) | 160 (29.0%) | 107 (23.7%) | 0.999 | 0.393 |
| Vasopressor Indicator | 21 (4.2%) | 27 (4.9%) | 22 (4.9%) | 0.999 | 0.999 |
| **Medications** |  |  |  |  |  |
| Heparin | 194 (38.5%) | 201 (36.5%) | 556 (53.9%) | 0.999 | 0.999 |
| Wafarin | 11 (2.2%) | 19 (3.4%) | 25 (2.4%) | 0.999 | 0.999 |
| Rivaroxaban | 17 (3.4%) | 13 (2.4%) | 16 (1.6%) | 0.999 | 0.999 |
| Dabigatran | 1 (0.2%) | 0 (0.0%) | 6 (0.6%) | 0.999 | 0.999 |
| Argatroban | 4 (0.8%) | 5 (0.9%) | 0 (0.0%) | 0.999 | 0.999 |
| Hydroxychloroquine | 320 (63.5%) | 356 (64.6%) | 29 (2.8%) | 0.999 | 0.311 |
| Azithromycin | 224 (44.4%) | 255 (46.3%) | 129 (12.5%) | 0.999 | 0.999 |
| Dexamethasone | 16 (3.2%) | 13 (2.4%) | 191 (18.5%) | 0.999 | 0.245 |
| Salicylic acid and derivatives | 130 (25.8%) | 133 (24.1%) | 368 (35.7%) | 0.999 | 0.584 |
| Ace inhibitor plain | 60 (11.9%) | 71 (12.9%) | 214 (20.7%) | 0.999 | 0.073 |
| Arb | 69 (13.7%) | 85 (15.4%) | 78 (17.3%) | 0.999 | 0.999 |
| **Vitals** |  |  |  |  |  |
| heart rate (beats/min) | 98.50 (23.09) | 100.60 (54.48) | 100.96 (56.043) | 0.999 | 0.999 |
| **Respiratory Measures** |  |  |  |  |  |
| Pulse Ox (%) | 95.00 (92.00, 97.00) | 95.00 (92.00, 97.00) | 95.00 (92.00, 97.00) | 0.999 | 0.999 |
| PaO2 (partial pressure of arterial oxygen, mm Hg) | 82.30 (63.40, 113.00) | 73.00 (63.00, 107.00) | 74.00 (62.00, 98.00) | 0.999 | 0.999 |
| FiO2 (fraction of inspired oxygen, %) | 50.00 (40.00, 50.00) | 50.00 (40.00, 60.00) | 50.00 (50.00, 100.00) | 0.999 | 0.067 |
| Osmolality (serum osmolality, mosm/Kg) | 292.00 (280.00, 317.00) | 286.00 (273.00, 311.00) | 291.00 (279.00, 315.00) | 0.999 | 0.999 |
| pH_arterial | 7.41 (7.34, 7.45) | 7.43 (7.36, 7.46) | 7.43 (7.36, 7.45) | 0.143 | 0.999 |
| O2_arterial (mm Hg) | 94.90 (92.00, 95.00) | 94.00 (91.00, 95.00) | 94.00 (91.00, 95.00) | 0.999 | 0.999 |
| CO2_arterial (mm Hg) | 38.00 (33.90, 46.00) | 36.50 (32.00, 43.00) | 37.00 (32.00, 47.00) | 0.762 | 0.999 |
| **Renal Labs** |  |  |  |  |  |
| BUN (blood urea nitrogen, mg/dL) | 16.00 (10.00, 28.00) | 15.00 (10.00, 24.00) | 16.00 (11.00, 24.00) | 0.441 | 0.924 |
| Sodium (serum sodium, meq/L) | 137.00 (134.00, 139.00) | 137.00 (134.00, 140.00) | 137.00 (134.00, 140.00) | 0.999 | 0.999 |
| K (serum potassium, meq/L) | 4.10 (3.80, 4.50) | 4.10 (3.80, 4.50) | 4.20 (3.80, 4.50) | 0.999 | 0.999 |
| Cl (serum chloride, meq/L) | 99.00 (95.00, 102.00) | 98.00 (95.00, 101.00) | 98.00 (95.00, 102.00) | 0.873 | 0.999 |
| hco3 (serum bicarbonate, meq/L) | 23.00 (21.00, 26.00) | 24.00 (21.00, 26.00) | 24.00 (21.00, 26.00) | 0.999 | 0.999 |
| Ca (serum calcium, mg/dL) | 9.00 (8.60, 9.30) | 9.10 (8.70, 9.40) | 9.10 (8.70, 9.50) | 0.056 | 0.482 |
| Ca_ionized (ionized calcium, mg/dL) | 4.50 (4.20, 4.70) | 4.50 (4.30, 4.70) | 4.50 (4.30, 4.70) | 0.999 | 0.999 |
| Phosphate (mg/dL) | 3.20 (2.70, 3.80) | 3.10 (2.70, 3.60) | 3.20 (2.60, 3.70) | 0.531 | 0.616 |
| First Mg (magnesium) | 2.00 (1.80, 2.20) | 2.00 (1.90, 2.20) | 2.00 (1.80, 2.20) | 0.999 | 0.999 |
| Na urine (urine sodium, meq/L) | 29.50 (16.00, 59.00) | 36.50 (19.00, 71.00) | 45.50 (21.00, 85.00) | 0.343 | 0.809 |
| Osmolality urine (mosm/Kg) | 451.00 (327.00, 560.00) | 482.00 (377.00, 604.50) | 401.50 (307.00, 537.00) | 0.999 | 0.2 |
| Creatinine (serum creatinine, mg/dL) | 0.91 (0.69, 1.31) | 0.89 (0.71, 1.15) | 0.91 (0.72, 1.17) | 0.999 | 0.999 |
| Creatinine urine (urine creatinine, mg/dL) | 105.97 (70.27, 169.71) | 100.94 (57.48, 165.14) | 87.03 (51.69, 151.28) | 0.999 | 0.416 |
| Urea urine (urine urea, mg/dL) | 421.00 (274.00, 707.00) | 531.00 (333.00, 716.00) | 521.00 (320.00, 771.00) | 0.999 | 0.999 |
| Protein urine strip (urine protein by dipstick) | 100.00 (30.00, 100.00) | 100.00 (30.00, 100.00) | 100.00 (30.00, 100.00) | 0.973 | 0.999 |
| Protein Urine (spot urine protein, mg/dL) | 50.40 (23.90, 93.40) | 45.70 (23.40, 96.70) | 41.40 (16.10, 102.00) | 0.999 | 0.999 |
| RBC urine (urine red blood cells) | 2.00 (1.00, 10.00) | 2.00 (1.00, 8.00) | 2.00 (1.00, 7.00) | 0.999 | 0.999 |
| Renin (serum renin, ng/mL/hr) | 2.30 (0.60, 12.30) | 1.60 (0.60, 4.55) | 0.70 (0.30, 2.25) | 0.745 | 0.013* |
| **Other Labs** |  |  |  |  |  |
| Lactate (mmol/L) | 1.40 (1.10, 2.00) | 1.40 (1.10, 1.90) | 1.60 (1.20, 2.10) | 0.999 | 0.198 |
| BNP (pg/mL) | 283.50 (64.00, 1264.0) | 167.50 (48.00, 847.00) | 276.00 (84.00, 1160.0) | 0.071 | 0.025* |
| Troponin (ng/mL) | 0.01 (0.01, 0.01) | 0.01 (0.01, 0.01) | 0.01 (0.01, 0.01) | 0.374 | 0.999 |
| INR | 1.20 (1.10, 1.30) | 1.20 (1.10, 1.30) | 1.10 (1.10, 1.30) | 0.083 | 0.669 |
| LDH (lactate dehydrogenase, U/L) | 284.00 (212.00, 385.00) | 309.50 (212.00, 411.50) | 268.50 (219.00, 335.00) | 0.999 | 0.999 |
| AST (aspartate aminotransferase, U/L) | 37.00 (25.00, 60.00) | 39.00 (26.50, 61.00) | 38.00 (25.00, 58.00) | 0.999 | 0.999 |
| ALT (alanine aminotransferase, U/L) | 29.00 (17.00, 44.00) | 30.00 (17.00, 51.00) | 27.00 (17.00, 53.00) | 0.641 | 0.999 |
| CPK (creatinine phosphokinase, U/L) | 92.50 (50.00, 232.00) | 107.00 (52.50, 234.00) | 96.00 (51.00, 216.50) | 0.999 | 0.999 |
| **Lipid Profile** |  |  |  |  |  |
| LDL (low density lipoprotein, mg/dL) | 61.00 (45.00, 87.00) | 70.00 (51.00, 93.00) | 71.00 (53.00, 92.00) | 0.411 | 0.999 |
| Triglyceride (mg/dL) | 127.00 (90.00, 182.00) | 126.50 (97.00, 189.50) | 125.00 (86.00, 182.00) | 0.999 | 0.999 |
| HDL (high density lipoprotein, mg/dL) | 32.00 (23.00, 37.00) | 32.50 (25.00, 40.50) | 33.00 (25.00, 43.00) | 0.741 | 0.999 |
|  |  |  |  |  |  |

**^#^CONTROL GROUP**

**Data were shown with n (%) for categorical variables, mean (sd) and median (interquartile range) for continuous variables.**

*** p<0.05; P values were based on ANOVA with Dunnett’s adjustment, Kruskal-Wallis test with DSCF adjustment and Chi-square test with Bonferroni adjustment for multiple comparisons.**

**p-value^1^**-T1 vs. T2

**p-value^2^**-T3 vs. T2
