# Supplementary material for: Initial mean arterial blood pressure (MABP) measurement is a risk factor for mortality in hypertensive COVID-19 positive hospitalized patients
Source: PLoS One. 2023 Mar 30;18(3):e0283331. doi: 10.1371/journal.pone.0283331 (PMC10062544; doi:10.1371/journal.pone.0283331)
Supplement: S3 Table — (DOCX) [file pone.0283331.s003.docx]

**S3 Table. Tertiles of MABP in COVID-19 (-) patients**

| **Variables** | **COVID-19 Negative** | | | | |
| --- | --- | --- | --- | --- | --- |
|  | **T1 ≥65- <86 mmHg** | **T2# ≥86-<98 mmHg** | **T3 ≥ 98 mmHg** | **p- value^1^** | **p-value^2^** |
|  | N=711 | N=772 | N=1032 |  |  |
| **Demographics** |  |  |  |  |  |
| Age | 54.64 (21.95) | 55.16 (21.95) | 61.35 (20.16) | 0.999 | <0.0001* |
| **Gender** |  |  |  |  |  |
| Female | 473 (57.8%) | 475 (57.6%) | 522 (49.3%) | 0.999 | <0.0001* |
| Male | 345 (42.2%) | 349 (42.4%) | 536 (50.7%) |  |  |
| **Race** |  |  |  |  |  |
| American Indian or Alaska Native | 2 (0.2%) | 0 (0.0%) | 0 (0.0%) | 0.999 | 0.999 |
| Asian | 22 (2.7%) | 20 (2.4%) | 27 (2.6%) |  |  |
| Black or African American | 51 (6.2%) | 40 (4.9%) | 80 (7.6%) |  |  |
| Other Race/Unknown | 128 (15.6%) | 106 (12.9%) | 108 (10.2%) |  |  |
| White | 615 (75.2%) | 658 (79.9%) | 843 (79.7%) |  |  |
| **Ethicity** |  |  |  |  |  |
| Hispanic or Latino | 46 (5.6%) | 40 (4.9%) | 47 (4.4%) | 0.999 | 0.415 |
| Not Hispanic | 772 (94.4%) | 784 (95.1%) | 1011 (95.6%) |  |  |
| **Comorbid conditions** |  |  |  |  |  |
| Diabetes mellitus | 146 (20.5%) | 152 (19.7%) | 234 (22.7%) | 0.999 | 0.884 |
| HF (heart failure) | 132 (18.6%) | 116 (15.0%) | 171 (16.6%) | 0.479 | 0.999 |
| CKD (chronic kidney disease) | 88 (12.4%) | 91 (11.8%) | 140 (13.6%) | 0.999 | 0.999 |
| COPD (chronic obstructive pulmonary disease) | 87 (12.2%) | 92 (11.9%) | 137 (13.3%) | 0.999 | 0.999 |
| HTN (hypertension) | 216 (30.4%) | 230 (29.8%) | 539 (52.2%) | 0.999 | <0.0001* |
| CAD (coronary artery disease) | 155 (21.8%) | 149 (19.3%) | 255 (24.7%) | 0.999 | 0.046* |
| Cancer | 125 (17.6%) | 128 (16.6%) | 116 (11.2%) | 0.999 | 0.008* |
| Asthma | 61 (8.6%) | 48 (6.2%) | 61 (5.9%) | 0.578 | 0.999 |
| Suicidal_Thoughts | 18 (2.5%) | 18 (2.3%) | 19 (1.8%) | 0.999 | 0.999 |
| Major_Depression | 122 (17.2%) | 99 (12.8%) | 143 (13.9%) | 0.137 | 0.999 |
| Schizophrenia | 6 (0.8%) | 8 (1.0%) | 14 (1.4%) | 0.999 | 0.999 |
| Bipolar | 28 (3.9%) | 31 (4.0%) | 44 (4.3%) | 0.999 | 0.999 |
| ADHD (attention deficit hyperactivity disorder) | 12 (1.7%) | 15 (1.9%) | 10 (1.0%) | 0.999 | 0.602 |
| Anxiety | 143 (20.1%) | 143 (18.5%) | 187 (18.1%) | 0.999 | 0.999 |
| Transplant_Liver | 0 (0.0%) | 0 (0.0%) | 1 (0.1%) | 0.999 | 0.999 |
| BMI | 27.78 (7.02) | 28.40 (6.74) | 28.12 (6.97) | 0.777 | 0.999 |
| **Severity of illness** |  |  |  |  |  |
| Length of Hospital Stay | 5.00 (3.00, 8.00) | 4.00 (3.00, 7.00) | 5.00 (3.00, 8.00) | 0.002* | 0.005* |
| Invasive vent days (invasive ventilation) | 4.00 (2.00, 7.00) | 3.00 (2.00, 9.00) | 4.00 (2.00, 11.00) | 0.999 | 0.999 |
| ICU Admission | 90 (12.7%) | 85 (11.0%) | 169 (16.4%) | 0.999 | 0.009* |
| Length of ICU stay | 4.00 (2.00, 7.00) | 3.00 (2.00, 7.00) | 4.00 (2.00, 9.00) | 0.999 | 0.999 |
| Sepsis | 90 (12.7%) | 69 (8.9%) | 64 (6.2%) | 0.149 | 0.2 |
| Vasopressor Indicator | 98 (13.8%) | 114 (14.8%) | 174 (16.9%) | 0.999 | 0.999 |
| **Medications** |  |  |  |  |  |
| Enoxaparin | 145 (20.4%) | 167 (21.6%) | 250 (24.2%) | 0.999 | 0.999 |
| Heparin | 280 (39.4%) | 320 (41.5%) | 556 (53.9%) | 0.999 | <0.0001* |
| Wafarin | 25 (3.5%) | 17 (2.2%) | 25 (2.4%) | 0.916 | 0.999 |
| Rivaroxaban | 13 (1.8%) | 9 (1.2%) | 16 (1.6%) | 0.999 | 0.999 |
| Dabigatran | 7 (1.0%) | 3 (0.4%) | 6 (0.6%) | 0.999 | 0.999 |
| Argatroban | 1 (0.1%) | 1 (0.1%) | 0 (0.0%) | 0.999 | 0.999 |
| Hydroxychloroquine | 20 (2.8%) | 25 (3.2%) | 29 (2.8%) | 0.999 | 0.999 |
| Azithromycin | 105 (14.8%) | 104 (13.5%) | 129 (12.5%) | 0.999 | 0.999 |
| Dexamethasone | 82 (11.5%) | 110 (14.2%) | 191 (18.5%) | 0.842 | 0.116 |
| Salicylic acid and derivatives | 186 (26.2%) | 207 (26.8%) | 368 (35.7%) | 0.999 | 0.0005* |
| Ace inhibitor plain | 70 (9.8%) | 82 (10.6%) | 214 (20.7%) | 0.999 | <0.0001* |
| Arb | 80 (11.3%) | 87 (11.3%) | 191 (18.5%) | 0.999 | 0.0002* |
| **Vitals** |  |  |  |  |  |
| SBP | 115.39 (17.70) | 129.58 (19.67) | 153.64 (27.04) | <0.0001* | <0.0001* |
| DBP | 67.57 (10.83) | 75.21 (10.62) | 85.70 (13.98) | <0.0001* | <0.0001* |
| PPD | 48.30 (14.57) | 54.36 (17.60) | 67.98 (23.21) | <0.0001* | <0.0001* |
| MABP | 78.17 (5.26) | 91.55 (3.41) | 111.62 (20.96) | <0.0001* | <0.0001* |
| heart rate (beats/min) | 92.62 (21.84) | 91.99 (21.13) | 101.81 (303.61) | 0.999 | 0.999 |
| oral temperature (^o^C) | 36.99 (2.35) | 36.88 (0.47) | 36.86 (0.39) | 0.675 | 0.999 |
| respiratory rate (respirations/min) | 18.54 (4.24) | 18.58 (6.01) | 19.67 (13.13) | 0.999 | 0.081 |
| **Respiratory Measures** |  |  |  |  |  |
| Pulse Ox (%) | 98.00 (96.00, 99.00) | 98.00 (96.00, 99.00) | 97.00 (96.00, 99.00) | 0.999 | 0.497 |
| PaO2 (partial pressure of arterial oxygen, mm Hg) | 98.00 (69.10, 181.00) | 95.00 (74.00, 153.00) | 95.50 (75.00, 178.00) | 0.999 | 0.999 |
| FiO2 (fraction of inspired oxygen, %) | 50.00 (40.00, 100.00) | 50.00 (40.00, 100.00) | 50.00 (40.00, 100.00) | 0.999 | 0.999 |
| Osmolality (serum osmolality, mosm/Kg) | 289.00 (278.00, 301.00) | 290.50 (274.50, 304.50) | 286.00 (271.00, 299.00) | 0.999 | 0.999 |
| pH_arterial | 7.37 (7.29, 7.43) | 7.38 (7.32, 7.42) | 7.39 (7.32, 7.42) | 0.999 | 0.999 |
| O2_arterial (mm Hg) | 95.00 (92.00, 95.30) | 95.00 (93.00, 95.00) | 95.00 (93.00, 95.00) | 0.999 | 0.999 |
| CO2_arterial (mm Hg) | 40.00 (35.10, 50.80) | 40.00 (35.10, 46.90) | 41.00 (36.00, 47.00) | 0.999 | 0.999 |
| **Renal Labs** |  |  |  |  |  |
| BUN (blood urea nitrogen, mg/dL) | 18.00 (13.00, 30.00) | 17.00 (11.00, 26.00) | 17.00 (12.00, 23.00) | 0.027* | 0.999 |
| Sodium (serum sodium, meq/L) | 137.00 (135.00, 140.00) | 138.00 (135.00, 140.00) | 138.00 (136.00, 140.00) | 0.622 | 0.51 |
| K (serum potassium, meq/L) | 4.20 (3.80, 4.60) | 4.10 (3.80, 4.50) | 4.20 (3.80, 4.50) | 0.999 | 0.999 |
| Cl (serum chloride, meq/L) | 100.00 (97.00, 103.00) | 101.00 (97.00, 103.00) | 101.00 (97.00, 103.00) | 0.999 | 0.999 |
| hco3 (serum bicarbonate, meq/L) | 24.00 (21.00, 26.00) | 24.00 (22.00, 26.00) | 24.00 (22.00, 26.00) | 0.999 | 0.999 |
| Ca (serum calcium, mg/dL) | 9.30 (8.90, 9.70) | 9.40 (9.00, 9.80) | 9.50 (9.10, 9.80) | 0.101 | 0.542 |
| Ca_ionized (ionized calcium, mg/dL) | 4.55 (4.20, 4.70) | 4.70 (4.50, 4.80) | 4.60 (4.40, 4.90) | 0.019* | 0.999 |
| Phosphate (mg/dL) | 3.40 (2.80, 4.00) | 3.40 (2.80, 3.90) | 3.20 (2.80, 3.80) | 0.999 | 0.185 |
| First Mg (magnesium) | 1.90 (1.70, 2.10) | 2.00 (1.80, 2.10) | 2.00 (1.80, 2.10) | 0.999 | 0.999 |
| Na urine (urine sodium, meq/L) | 37.00 (20.00, 67.00) | 56.00 (30.00, 90.00) | 61.00 (36.00, 102.00) | 0.005* | 0.999 |
| Osmolality urine (mosm/Kg) | 388.00 (292.00, 477.00) | 435.50 (316.50, 550.50) | 421.50 (352.00, 526.00) | 0.941 | 0.999 |
| Creatinine (serum creatinine, mg/dL) | 0.94 (0.74, 1.36) | 0.90 (0.70, 1.21) | 0.90 (0.70, 1.16) | 0.056 | 0.999 |
| Creatinine urine (urine creatinine, mg/dL) | 75.34 (51.64, 135.94) | 77.03 (48.70, 112.73) | 71.95 (42.88, 117.20) | 0.999 | 0.999 |
| Urea urine (urine urea, mg/dL) | 504.50 (384.50, 797.50) | 529.50 (350.00, 741.00) | 399.00 (217.00, 680.00) | 0.999 | 0.495 |
| Protein urine strip (urine protein by dipstick) | 30.00 (30.00, 100.00) | 100.00 (30.00, 100.00) | 100.00 (30.00, 100.00) | 0.999 | 0.999 |
| Protein Urine (spot urine protein, mg/dL) | 24.40 (12.70, 68.80) | 31.80 (15.70, 69.40) | 26.70 (12.90, 74.00) | 0.999 | 0.999 |
| RBC urine (urine red blood cells) | 2.00 (1.00, 7.00) | 2.00 (1.00, 8.00) | 1.00 (1.00, 5.00) | 0.999 | 0.999 |
| Renin (serum renin, ng/mL/hr) | 3.95 (1.05, 8.90) | 1.30 (0.60, 2.70) | 0.85 (0.30, 2.60) | 0.283 | 0.999 |
| **Inflammatory Labs** |  |  |  |  |  |
| Ferritin (mcg/L) | 206.10 (85.00, 535.70) | 223.30 (76.10, 524.20) | 184.30 (88.50, 398.50) | 0.999 | 0.999 |
| Albumin Serum (mg/dL) | 3.80 (3.40, 4.20) | 3.90 (3.50, 4.30) | 4.10 (3.70, 4.40) | 0.005* | <0.0001* |
| Lymphocyte Count (K/mm^3^) | 1.31 (0.82, 2.00) | 1.36 (0.89, 2.00) | 1.45 (0.97, 2.03) | 0.999 | 0.703 |
| Procalcitonin (ng/mL) | 0.19 (0.09, 0.87) | 0.13 (0.08, 0.33) | 0.10 (0.06, 0.23) | 0.026* | 0.013* |
| D-Dimer (ng/mL) | 506.00 (293.00, 1184.0) | 451.00 (230.00, 1132.0) | 371.00 (204.00, 895.50) | 0.495 | 0.653 |
| IL6 (pg/mL) | 8.35 (4.90, 91.25) | 30.40 (6.40, 69.40) | 11.90 (4.75, 36.50) | 0.999 | 0.999 |
| WBC (10^9^ cell/L) | 10.24 (7.47, 13.81) | 9.96 (7.43, 12.91) | 9.49 (7.38, 12.34) | 0.999 | 0.999 |
| ESR (mm/hr) | 41.00 (17.50, 70.50) | 29.00 (14.00, 59.00) | 22.00 (13.00, 52.00) | 0.999 | 0.999 |
| CRP (mg/L) | 3.70 (0.80, 10.10) | 3.00 (0.70, 7.65) | 1.50 (0.40, 6.20) | 0.242 | 0.118 |
| **Other Labs** |  |  |  |  |  |
| HB (g/dL) | 12.20 (10.50, 13.60) | 12.70 (11.20, 14.00) | 13.20 (11.80, 14.60) | 0.0002* | <0.0001* |
| Lactate (mmol/L) | 1.60 (1.10, 2.60) | 1.50 (1.10, 2.25) | 1.50 (1.10, 2.30) | 0.445 | 0.999 |
| BNP (pg/mL) | 861.00 (201.00, 3512.0) | 881.00 (213.00, 3018.0) | 490.00 (128.00, 2185.0) | 0.999 | 0.069 |
| Troponin (ng/mL) | 0.01 (0.01, 0.03) | 0.01 (0.01, 0.02) | 0.01 (0.01, 0.01) | 0.804 | 0.113 |
| INR | 1.20 (1.10, 1.40) | 1.10 (1.00, 1.30) | 1.10 (1.00, 1.20) | 0.0002* | <0.0001* |
| LDH (lactate dehydrogenase, U/L) | 238.00 (189.00, 332.00) | 216.00 (186.00, 288.00) | 234.00 (192.00, 300.00) | 0.999 | 0.999 |
| AST (aspartate aminotransferase, U/L) | 25.00 (18.00, 40.00) | 24.00 (18.00, 40.00) | 24.00 (18.00, 36.00) | 0.999 | 0.999 |
| ALT (alanine aminotransferase, U/L) | 21.00 (13.00, 34.00) | 19.00 (13.00, 33.00) | 19.00 (13.00, 33.00) | 0.999 | 0.999 |
| CPK (creatinine phosphokinase, U/L) | 84.50 (46.00, 218.00) | 102.50 (50.00, 343.00) | 142.00 (64.00, 430.00) | 0.999 | 0.441 |
| **Lipid Profile** |  |  |  |  |  |
| LDL (low density lipoprotein, mg/dL) | 66.00 (45.00, 94.00) | 75.00 (53.00, 96.50) | 83.00 (62.00, 116.00) | 0.667 | 0.002* |
| Triglyceride (mg/dL) | 108.00 (76.00, 157.00) | 97.00 (73.00, 145.00) | 102.00 (73.00, 167.00) | 0.999 | 0.999 |
| HDL (high density lipoprotein, mg/dL) | 38.00 (29.00, 50.00) | 44.00 (32.00, 55.00) | 46.00 (37.00, 59.00) | 0.007* | 0.076 |
| **death** | **18(2.5)** | **30(3.9)** | **23(2.2)** | 0.999 | 0.292 |

**^#^CONTROL GROUP**

**Data were shown with n (%) for categorical variables, mean (sd) and median (interquartile range) for continuous variables.**

*** p<0.05; P values were based on ANOVA with Dunnett’s adjustment, Kruskal-Wallis test with DSCF adjustment and Chi-square test with Bonferroni adjustment for multiple comparisons.**

**p-value^1^**-T1 vs. T2

**p-value^2^**-T3 vs. T2
