# Supplementary material for: Initial mean arterial blood pressure (MABP) measurement is a risk factor for mortality in hypertensive COVID-19 positive hospitalized patients
Source: PLoS One. 2023 Mar 30;18(3):e0283331. doi: 10.1371/journal.pone.0283331 (PMC10062544; doi:10.1371/journal.pone.0283331)
Supplement: S4 Table — (DOCX) [file pone.0283331.s004.docx]

**S4 Table. Comparison of hypertensive COVID-19 positive patients who died and survived.**

| **Variables** | **Mortality Status** | | |
| --- | --- | --- | --- |
|  | **Live** | **Dead** | **P-value** |
| **N =627** | **N=538** | **N=89** |  |
| **Comorbid conditions** |  |  |  |
| HF (heart failure) | 42 (7.8%) | 10 (11.2%) | 0.277 |
| Cancer | 41 (7.6%) | 8 (9.0%) | 0.656 |
| Asthma | 43 (8.0%) | 3 (3.4%) | 0.121 |
| Suicidal Thoughts | 5 (0.9%) | 0 (0.0%) | 0.361 |
| Major Depression | 92 (17.1%) | 11 (12.4%) | 0.264 |
| Schizophrenia | 7 (1.3%) | 1 (1.1%) | 0.89 |
| Bipolar | 9 (1.7%) | 3 (3.4%) | 0.279 |
| ADHD (attention deficit hyperactivity disorder) | 1 (0.2%) | 1 (1.1%) | 0.146 |
| Anxiety | 88 (16.4%) | 9 (10.1%) | 0.131 |
| BMI | 28.18 (7.53) | 28.52 (7.08) | 0.705 |
| **Severity of illness** |  |  |  |
| Length of Hospital Stay (days) | 8.00 (5.00, 13.00) | 13.00 (7.00, 25.00) | 0.0002* |
| Invasive vent days (invasive ventilation) | 15.00 (10.00, 31.00) | 13.00 (7.00, 23.00) | 0.124 |
| ICU Admission | 88 (16.4%) | 56 (62.9%) | <0.0001* |
| Length of ICU stay (days) | 12.00 (4.50, 26.50) | 11.50 (7.00, 20.50) | 0.636 |
| Sepsis | 137 (25.5%) | 56 (62.9%) | <0.0001* |
| Vasopressor Indicator | 26 (4.8%) | 4 (4.5%) | 0.89 |
| **Medications** |  |  |  |
| Enoxaparin | 382 (71.0%) | 68 (76.4%) | 0.294 |
| Heparin | 208 (38.7%) | 54 (60.7%) | <0.0001* |
| Warfarin | 20 (3.7%) | 2 (2.2%) | 0.485 |
| Rivaroxaban | 20 (3.7%) | 1 (1.1%) | 0.208 |
| Dabigatran | 0 (0.0%) | 0 (0.0%) | 0.208 |
| Argatroban | 2 (0.4%) | 3 (3.4%) | 0.003* |
| Hydroxychloroquine | 361 (67.1%) | 70 (78.7%) | 0.029* |
| Azithromycin | 239 (44.4%) | 51 (57.3%) | 0.024* |
| Dexamethasone | 18 (3.3%) | 2 (2.2%) | 0.585 |
| Salicylic acid and derivatives | 176 (32.7%) | 34 (38.2%) | 0.31 |
| Ace inhibitor plain | 138 (25.7%) | 16 (18.0%) | 0.119 |
| Arb | 151 (28.1%) | 21 (23.6%) | 0.381 |
| **Respiratory Measures** |  |  |  |
|  |  |  |  |
| PaO2 (partial pressure of arterial oxygen, mm Hg) | 73.00 (61.00, 97.00) | 71.50 (56.00, 105.00) | 0.522 |
| FiO2 (fraction of inspired oxygen, %) | 50.00 (40.00, 50.00) | 50.00 (50.00, 100.00) | 0.0004* |
| Osmolality (serum osmolality, mosm/Kg) | 289.00 (275.00, 310.00) | 293.00 (281.00, 318.00) | 0.408 |
| pH_arterial | 7.43 (7.38, 7.46) | 7.41 (7.31, 7.46) | 0.081 |
| O2_arterial (mm Hg) | 94.00 (91.50, 95.00) | 93.00 (88.00, 95.00) | 0.038* |
| CO2_arterial (mm Hg) | 37.00 (32.00, 41.85) | 39.00 (33.00, 48.60) | 0.040* |
| **Renal Labs** |  |  |  |
| Sodium (serum sodium, meq/L) | 137.00 (134.00, 139.00) | 137.00 (132.00, 139.00) | 0.522 |
| Cl (serum chloride, meq/L) | 98.00 (95.00, 101.00) | 97.00 (94.00, 102.00) | 0.539 |
| Ca_ionized (ionized calcium, mg/dL) | 4.55(0.45) | 4.38(0.46) | 0.0421* |
| Phosphate (mg/dL) | 3.10 (2.60, 3.60) | 3.10 (2.60, 3.80) | 0.314 |
| First Mg (magnesium) | 2.00 (1.80, 2.20) | 2.00 (1.80, 2.20) | 0.201 |
| Na urine (urine sodium, meq/L) | 36.00 (17.00, 65.00) | 28.00 (15.00, 58.00) | 0.117 |
| Osmolality urine (mosm/Kg) | 448.00 (354.00, 598.00) | 412.00 (327.00, 528.00) | 0.328 |
| Creatinine urine (urine creatinine, mg/dL) | 107.64 (66.83, 167.81) | 100.00 (77.13, 161.25) | 0.616 |
| Urea urine (urine urea, mg/dL) | 530.00 (344.00, 825.00) | 291.00 (218.00, 716.00) | 0.079 |
| Protein urine strip (urine protein by dipstick) | 100.00 (30.00, 100.00) | 100.00 (30.00, 100.00) | 0.107 |
| Protein Urine (spot urine protein, mg/dL) | 47.90 (20.10, 88.80) | 78.50 (42.40, 114.40) | 0.023* |
| RBC urine (urine red blood cells) | 1.00 (1.00, 5.00) | 3.00 (1.00, 13.00) | 0.003* |
| Renin (serum renin, ng/mL/hr) | 1.45 (0.50, 5.60) | 0.85 (0.50, 8.20) | 0.87 |
| **Inflammatory Labs** |  |  |  |
| Procalcitonin (ng/mL) | 0.14 (0.09, 0.25) | 0.26 (0.16, 0.54) | <0.0001* |
| IL6 (pg/mL) | 44.70 (17.40, 82.00) | 103.50 (53.70, 229.00) | <0.0001* |
| ESR (mm/hr) | 54.00 (30.00, 76.00) | 63.00 (44.00, 87.00) | 0.013* |
| CRP (mg/L) | 6.80 (2.60, 13.40) | 13.40 (7.50, 21.90) | <0.0001* |
| **Other Labs** |  |  |  |
| HB (g/dL) | 13.20 (11.80, 14.40) | 13.10 (11.90, 14.70) | 0.664 |
| Lactate (mmol/L) | 1.40 (1.10, 2.00) | 1.90 (1.50, 2.80) | <0.0001* |
| BNP (pg/mL) | 214.50 (66.00, 705.00) | 1123.0 (373.00, 2461.0) | <0.0001* |
| Troponin (ng/mL) | 0.01 (0.01, 0.01) | 0.01 (0.01, 0.03) | <0.0001* |
| INR | 1.10 (1.10, 1.30) | 1.20 (1.10, 1.30) | 0.045* |
| LDH (lactate dehydrogenase, U/L) | 253.00 (209.00, 342.00) | 411.00 (310.00, 547.00) | 0.0003* |
| AST (aspartate aminotransferase, U/L) | 37.00 (25.00, 56.00) | 54.50 (33.50, 76.00) | <0.0001* |
| ALT (alanine aminotransferase, U/L) | 29.00 (17.00, 43.00) | 30.00 (20.50, 52.50) | 0.131 |
| CPK (creatinine phosphokinase, U/L) | 97.00 (50.00, 230.00) | 139.00 (59.00, 469.00) | 0.015* |
| **Lipid Profile** |  |  |  |
| LDL (low density lipoprotein, mg/dL) | 71.00 (52.00, 90.50) | 61.00 (45.00, 84.00) | 0.279 |
| Triglyceride (mg/dL) | 121.00 (93.00, 186.00) | 155.00 (125.00, 241.00) | 0.001* |
| HDL (high density lipoprotein, mg/dL) | 32.00 (24.00, 40.00) | 31.00 (22.00, 38.00) | 0.448 |
|  |  |  |  |

**Data were shown with n (%) for categorical variables, mean (sd) and median (interquartile range) for continuous variables.**

*** p<0.05; P values were based on Chi-square tests, t-tests and Mann-Whitney tests.**
