# Supplementary material for: Initial mean arterial blood pressure (MABP) measurement is a risk factor for mortality in hypertensive COVID-19 positive hospitalized patients
Source: PLoS One. 2023 Mar 30;18(3):e0283331. doi: 10.1371/journal.pone.0283331 (PMC10062544; doi:10.1371/journal.pone.0283331)
Supplement: S5 Table — (DOCX) [file pone.0283331.s005.docx]

**S5 Table. Comparison of non-hypertensive COVID-19 positive patients who died and survived.**

| **Variables** | **Mortality Status** | | |
| --- | --- | --- | --- |
|  | **Live** | **Dead** | **P-value** |
|  | **N=801** | **N=121** |  |
| **Vitals** |  |  |  |
| SBP | 125.24 (22.18) | 121.21 (26.98) | 0.121 |
| DBP | 74.41 (13.10) | 69.23 (13.76) | 0.0002* |
| MAP | 89.77 (13.93) | 87.05 (16.78) | 0.094 |
|  |  |  |  |
| **Respiratory Measures** |  |  |  |
| pH_arterial | 7.43 (7.37, 7.46) | 7.37 (7.25, 7.42) | <0.0001* |
| O2_arterial (mm Hg) | 95.00 (93.00, 95.00) | 93.00 (88.90, 95.00) | 0.0003* |
| CO2_arterial (mm Hg) | 37.00 (32.00, 44.00) | 43.00 (32.00, 56.00) | 0.004* |
| **Renal Function** |  |  |  |
| Osmolality (serum osmolality, mosm/Kg) | 286.00 (276.00, 311.00) | 314.00 (291.00, 322.00) | 0.012* |
| Na urine (urine sodium, meq/L) | 43.00 (20.00, 80.00) | 28.00 (18.00, 51.00) | 0.021* |
| Osmolality urine (mosm/Kg) | 451.00 (355.00, 593.00) | 423.50 (315.50, 522.50) | 0.46 |
| Creatinine (serum creatinine, mg/dL) | 0.85 (0.66, 1.10) | 1.38 (0.98, 1.78) | <0.0001* |
| Creatinine urine (urine creatinine, mg/dL) | 89.63 (54.47, 146.72) | 119.41 (56.93, 176.10) | 0.256 |
| Urea urine (urine urea, mg/dL) | 549.00 (323.00, 764.00) | 410.00 (237.00, 597.00) | 0.105 |
| Protein urine strip (urine protein by dipstick) | 100.00 (30.00, 100.00) | 100.00 (30.00, 100.00) | 0.028* |
| Protein Urine (spot urine protein, mg/dL) | 37.20 (16.10, 93.20) | 81.85 (43.15, 146.05) | 0.001* |
| RBC urine (urine red blood cells) | 2.00 (1.00, 7.00) | 6.00 (1.00, 22.00) | <0.0001* |
| Renin (serum renin, ng/mL/hr) | 1.45 (0.50, 4.90) | 2.70 (0.65, 25.00) | 0.08 |
|  |  |  |  |

**Data were shown with n (%) for categorical variables, mean (sd) and median (interquartile range) for continuous variables.**

*** p<0.05; P values were based on Chi-square tests, t-tests and Mann-Whitney tests.**
